# Age matters: exploring differential effects of antimicrobial treatment on gut microbiota of adult and juvenile brown trout (*Salmo trutta*)

**DOI:** 10.1186/s42523-025-00391-2

**Published:** 2025-03-16

**Authors:** Lisa-Marie Streb, Paulina Cholewińska, Silvia Gschwendtner, Juergen Geist, Susanne Rath, Michael Schloter

**Affiliations:** 1 Research Unit Comparative Microbiome Analysis, Helmholtz Munich, Neuherberg, Germany; 2 Chair for Fish Diseases and Fisheries Biology, Ludwig-Maximilian University, Munich, Germany; 3 TUM School of Life Sciences, Chair of Aquatic Systems Biology, Technical University Munich, Freising, Germany; 4 Institute for Chemistry, University of Campinas, Campinas, Brazil; 5 TUM School of Life Sciences, Chair of Environmental Microbiology, Technical University Munich, Freising, Germany

**Keywords:** Florfenicol, Peracetic acid, Antibiotics, Aquaculture, Gut microbiome, Host age

## Abstract

**Background:**

Antibiotics and antiparasitics are essential tools in controlling infectious disease outbreaks in commercial aquaculture. While the negative effects of antimicrobials on the gut microbiome of various farmed fish species are well documented, the influence of underlying host factors, such as age, on microbiome responses remains poorly understood. This is especially evident for peracetic acid, whose impact on the gut microbiome has not yet been studied. Understanding how microbiome dynamics vary by host age is critical to improving antibiotic stewardship in aquaculture. In this study, juvenile and sexually mature brown trout (*Salmo trutta*) were used as a model to investigate the age-dependent effects of florfenicol and peracetic acid on the gut microbiome using a 16S rRNA metabarcoding approach.

**Results:**

Fish age significantly shaped taxonomic composition and microbial co-occurrence network structure of the gut microbiome, regardless of treatment. Juvenile trout exhibited greater microbiome volatility and a stronger response to both florfenicol and peracetic acid compared to adult fish, with disruptions persisting up to 11 days post-treatment. Temporal dynamics were also evident, with microbial shifts characterized by a decline in beneficial commensals like *Cetobacterium* and *Lactococcus*. Although overall abundance recovered by 18 days post-treatment, network positions of key microbial community members remained altered, particularly in juvenile fish. Opportunistic pathogens, including Aeromonas and Streptococcus, were enriched and assumed more central roles within the microbial networks in treated fish.

**Conclusion:**

The initial composition of the gut microbiome in brown trout is strongly influenced by fish age, which in turn affects the microbiome’s response to antibiotic disruption. Juveniles displayed higher susceptibility to microbiome perturbation, and although recovery was observed at the community level, network properties remained altered. This study also provides the first evidence that external peracetic acid application can disrupt gut microbial communities. Since compositional shifts are often linked to functional alterations, even short-term disruptions may have important consequences for host health in developing fish. These findings emphasize the importance of considering gut microbial community structure in relation to fish age in aquaculture management practices.

**Supplementary Information:**

The online version contains supplementary material available at 10.1186/s42523-025-00391-2.

## Background

Ensuring food security in light of a growing world population and climate change is one of the defining challenges of our era. With a global production value of 185 million tons in 2022, worth approximately $ 296 billion, aquaculture is the fastest growing food sector and critical for economic development [1]. Salmonids such as Atlantic salmon (*Salmo salar*) and various trout species (e.g. *Oncorhynchus mykiss, Salmo trutta*) are of significant economic importance and widely farmed for both consumption and stocking purposes [2]. Given the reliance of aquaculture on healthy, fast-growing fish for achieving high production outputs and securing animal welfare, the significance of host-associated gut microbiota for overall fish health is increasingly recognized as a key factor for sustainable farming practices. The gut microbiota is integral to nutrient metabolism, mediation of immune homeostasis, and colonization resistance against pathogens, all of which are essential for the optimal development of fish. The intestinal microbiota in teleost fish is dominated by *Proteobacteria*, *Bacteroidota*, *Firmicutes* and *Fusobacteria*, although significant inter-specific variation is common [3]. Research has shown that the intestinal microbiota is modulated by a wide range of external factors such as diet [4, 5], water temperature [6], salinity [7], management practices [8] and geographical location [9], as well as internal factors including host physiology [10]. Specifically, host age and the associated physiochemical changes within the gut ecosystem during ontogeny, niche availability, and shifts in microbial interactions act as strong selective forces shaping the gut microbiome structure [11–15]. However, the gastrointestinal microbiota is highly vulnerable to antibiotic disruption, which is particularly concerning in aquaculture where disease outbreaks lead to significant economic losses [1]. As a result, the sector relies heavily on antimicrobials for pathogen control. Florfenicol, a fluorinated derivate of chloramphenicol, is a commonly used antibiotic in fish farming due to its broad spectrum efficacy and has been successfully applied for decades against infections like furunculosis (*Aeromonas salmonicida*) [16], enteric redmouth disease (*Yersinia ruckeri*) [17] and rainbow trout fry syndrome, a septicemic infection of juvenile trout caused by *Flavobacterium psychrophilum* [18]. With rising water temperatures accelerating the prevalence of ectoparasitic infections, addressing both bacterial and parasitic threats has become increasingly critical in aquaculture, necessitating the combined use of different compounds to efficiently counteract co-infections [19, 20]. Even at low doses, Peracetic Acid (PAA) is a promising alternative to traditional anti-parasitic compounds due to its low environmental toxicity and rapid degradation into acetic acid, oxygen, and water, leaving no toxic residues. Due to its strong antimicrobial properties through oxidation, disruption of cell membranes and protein denaturation, PAA effectively controls water-borne ectoparasites like *Ichthyophthirius multifiliis* and *Trichodina spp.* [21, 22] and fish pathogens including *Aeromonas sp.* and *Vibrio sp*. [23, 24]. Disease control is crucial in high-density rearing environments like hatcheries as juvenile fish are more vulnerable to contracting infections due to their developing immune system. Younger host age typically correlates with higher infection rates and severity of clinical symptoms [25–27]. Effective management practices, including the use of antibiotics like florfenicol and disinfectants like PAA, are essential to reducing mortality rates and enhancing growth, contributing to sustainable aquaculture by increasing production outputs.

Broad-spectrum antibiotics are frequently linked to severe dysbiosis within host-associated microbiomes through eradication of both pathogenic and commensal populations, often with deleterious consequences for the host [28, 29]. Observed changes often include (functional) shifts in global microbiome structure, characterized by a decreased abundance of beneficial taxa like *Cetobacterium* and *Pediococcus* and changes of *Proteobacteria:Bacteroidota* and *Proteobacteria:Firmicutes* ratios, signatures typically linked to dysbiosis [30–34]. Simultaneously, most studies observed significant increases of opportunistic pathogens (e.g. *Aeromonas, Shewanella* and *Citrobacter*) during treatment and enrichment of antibiotic resistance genes (ARG) and mobile genetic elements (MGE) [29, 34, 35]. Except for two recent studies by Galgano et al*.* [36, 37] on PAA exposure affecting the gut microbiome and AMR profiles of broilers and pigs, no research has investigated the impact of external PAA on the gut microbiota of fish to date. In the context of aquaculture, most studies investigating PAA have focused on bactericidal effects on a single-strain level or on microbiomes associated with rearing water or biofilters [38–41]. Co-application of florfenicol and peracetic acid may have a synergistic effect, potentially exacerbating disturbances on the community level as well as co-selection of antibiotic resistance genes (ARG) [42, 43]. However, the manner in which the individual microbiome responds to antibiotics and biocides, whether acute or chronic, depends on the initial state of the community and the intensity of exposure. Key determinants influencing the stability of the microbial community and its capacity to recover from external disruptions include host age, environment, health status and diet [29, 44–46]. As known from large-scale human and mouse studies, a mature microbiome can rebound faster and resist disruptions more effectively than a highly volatile, maturing microbiome, leading to more drastic changes in the latter [47–49]. In humans, the critical window for antibiotic-induced shifts is early childhood (age 0–3 years), a period of immense community turnover following initial colonization until a stable population equilibrium is reached. Given the evidence on the role of host age in microbiome assembly across different fish species and the link between disease susceptibility and age, similar patterns are likely present in fish. Frequent exposure to antibiotics during this critical phase might disrupt microbiome stability, fostering the spread of ARGs and, in the long run, increasing the susceptibility to infections by weakening host fitness. Still, age-dependent effects of antibiotic treatment remain understudied in fish. Most studies have focused on single compounds, rather than on the combined use of antibiotics with disinfectants like PAA, without including co-variables like host age, thus only reflecting a fragment of the variability in potential antibiotic effects. Understanding the factors that shape the microbiome response under external stress is vital for informed policy measures and good antibiotic stewardship in commercial fish farming.

Our study expanded the current knowledge by investigating age-related differences in the gut microbiome response to peracetic acid and florfenicol, individually and in combination, of adult and juvenile brown trout (*Salmo trutta*) using a 16S rRNA metabarcoding approach. Notably, this is the first study to assess the impact of peracetic acid on fish gut microbiome composition, incorporating a time series with an extended post-treatment phase to evaluate the reversibility of microbiome alterations following antimicrobial exposure. We hypothesized that under identical rearing conditions, juvenile trout with their maturing microbiome, would exhibit a stronger and prolonged response due to increased vulnerability. Additionally, based on previous evidence of additive effects with co-administered antibiotics on ARG dynamics, we excepted similar additive effects on the gut microbiome composition in the combined treatment group. External peracetic acid, on the other hand, is not expected to induce substantial changes in gut microbiome composition and diversity.

## Material and methods

### Experimental design and animal handling

In a seven-week trial, the age-dependent effects of florfenicol and peracetic acid application on the gut microbiome of brown trout (*Salmo trutta*) in a recirculating aquaculture system (RAS) were evaluated. The RAS was set up at the Aquatic Systems Biology Unit, Technical University Munich (Freising, Germany) from November 2021 to March 2022 by experienced personnel, following good veterinary practices. The setup was additionally evaluated and approved by the TUM Animal Welfare Office in accordance with existing regulations. All fish originated from the Institute’s breeding stock and had no prior exposure to antibiotics. Juvenile trout were one year old, whereas adult trout had already reached sexual maturation (~ 2 years old). Average weights (± SD) were 258.87 ± 71.17 g for adult and 34.21 ± 11.47 g for juvenile fish, with average sizes (± SD) of 27.82 ± 2.63 cm and 13.78 ± 1.63 cm, respectively (See Supplementary Table S1, Additional File 1). Following common farming practice, male and female adult brown trout as well as fingerlings were randomly distributed to eight 1.7 m^3^ plastic tanks at a stocking density of approximately eighty fish per tank, separated by age and treatment. The RAS received a constant freshwater influx of 2 l/min und was kept under a 12:12 h light:dark cycle. To reduce excess removal of florfenicol and peracetic acid residues, water exchange was minimized and limited to maintenance-related losses. Tanks were equipped with metal grids to collect debris and uneaten feed, with daily removal of excrements to avoid spoilage.

Daily monitoring of water quality parameters (dissolved oxygen: 8.76 ± 1.24 mg/L, temperature: 11.04 ± 0.18 °C, pH: 8.08 ± 0.12) was conducted using a WTW Multi 3040 Device (see Additional File 1: Table S2). Levels of harmful ammonium-N (NH_4_^+^-N) were measured daily using the Spectroquant Ammonium Reagent Test (0.41 ± 0.21 mg/L; Supelco, Sigma-Aldrich, USA).

The fish underwent a three-week acclimation period before the start of the experiment, during which they were fed with age-appropriate commercial diets (see Supplementary Table S3, Additional File 1) delivered by automated feeders operating from 8 am to 4 pm at a feeding rate of 1% body weight.

### Treatment and sampling

The study design comprised four treatment groups per age: (1) unmedicated control group, (2) antibiotic treatment with florfenicol, (3) antiparasitic treatment with peracetic acid (PAA) and (4) combined antibiotic and antiparasitic treatment with florfenicol and PAA. Florfenicol (Cayman Chemical, USA), dissolved in rapeseed-oil, was administered via top-coated feed for ten consecutive days at a dosage of 10 mg/kg body weight. Peracetic acid (0.005% v/v; Wolfasteril Classic©, Kesla Hygiene AG, Germany) was applied to the water surface on days one, three, six and nine during the treatment phase. Control groups received unmedicated feed coated with rapeseed oil during the treatment period to ensure comparability.

Fecal samples were collected at 5 sampling timepoints at least 3 h post feeding: day 0 (pre-treatment), day 10 (last day of treatment), day 14 (4 days post-treatment), day 21 (11 days post-treatment) and day 28 (18 days post-treatment). Fish were euthanized and the intestines were aseptically removed, and fecal material from the terminal part of the gut was then collected by gentle squeezing. Fecal material is a commonly used proxy in fish gut microbiome research and has been shown to provide a reliable representation of intestinal microbial communities despite certain limitations, which are discussed in detail below [50–52]. Whole gut tissue sampling, while possible, is prone to contamination and often results in co-amplification of host DNA, which can reduce the accuracy of microbial community profiling during amplicon sequencing [53–55]. This is particularly problematic in low biomass samples, such as small-sized juvenile fish gut. Given these considerations, fecal material was the most practical and reliable option for characterizing the gut microbiota in both juvenile and adult fish.

Five biological replicates were collected per timepoint, treatment and age group. Samples were immediately snap-frozen on dry ice and stored at − 20 °C until further processing.

### DNA extraction and 16S rRNA amplicon sequencing

Total DNA from 200 samples was extracted using the QIAamp Fast DNA Stool Mini Kit (QIAGEN, Netherlands) following the pathogen detection protocol of the manufacturer. Negative extraction controls were included to monitor potential sources of DNA contamination during the extraction process. DNA concentration was quantified using Quant-It™ PicoGreen dsDNA Assay Kit (ThermoFisher Scientific, USA), DNA purity was assessed spectrophotometrically with a Nano Drop 1000 device (ThermoFisher Scientific, USA).

Compositional shifts in microbial communities in response to antimicrobial treatment were assessed by high throughput amplicon sequencing targeting the V1-V2 region of the 16S rRNA gene. To minimize host DNA amplification, the primer pair 008F (5′-AGAGTTTGATCMTGGC-3′) and 343R (5’-CTGCTGCCTYCCGTA-3’) including overhang adaptors for index primers was used [56]. Preliminary tests demonstrated reduced off-target amplification of host DNA compared to the more commonly used 515F/806R primer set (Data not shown; [57, 58]).

PCR was performed in unicates in a 25 μL reaction mixture containing 80 ng DNA template, 12.5 μL NEBNext High-Fidelity Polymerase (New England Biolabs, USA) and 0.5 μL of each primer (10 pmol/μl). PCR conditions included an initial denaturation at 98 °C for 5 min, followed by 32 cycles of 10 s denaturation at 98 °C, 30 s annealing at 60 °C and 30 s extension at 72 °C and a final extension step for 5 min at 72 °C. Non-template samples and extraction controls were included to monitor the amplification of contaminant DNA. Quality of PCR products was assessed with gel electrophoresis and then purified using MagSi NGSprep Plus beads (Steinbrenner, Germany). Incorporation of Illumina indices from the Nextera XT Index Kit v2 (Illumina, Inc., USA) was done by PCR amplification with 12.5 µl NEBNext High Fidelity Master Mix, 2.5 µL of each primer and 10 ng of DNA template with the following conditions: 30 s at 98 °C, 10 cycles of 10 s at 98 °C, 30 s at 55 °C, 30 s at 72 °C, followed by 5 min at 72 °C. Quantity and quality of purified DNA libraries and the presence of primer dimers was checked on a fragment analyser (Agilent Technology, USA) using DNF-473 Standard Sensitivity NGS Fragment Kit. Due to insufficient yield or quality, 15 samples were excluded from the library preparation. Samples were pooled in an equimolar ratio of 4 nM and paired-end sequencing was performed on an Illumina MiSeq (2 × 300 bp) with reagent Kit v3 (600 cycles). Raw Sequence data is deposited in NCBI under BioProject ID PRJNA1162705.

### Bioinformatic processing

Bioinformatic processing of raw sequencing data and downstream statistical analysis was performed in R Studio (R Version 4.3.2; [59]). In total, 14,384,018 reads were obtained from 185 individuals. After demultiplexing, adapter sequences were removed using cutadapt v4.5 [60], following quality assessment with fastQC v0.12.1 [61]. Removal of primer sequences, trimming of low-quality bases and inference of amplicon sequencing variants (ASV) was done with *DADA2* v1.30.0 [62] with the following trimming parameters: n-terminal trimming of 20 bp, truncation at the forward position 240 (adult) and 230 (juvenile) and reverse positions 200 and 190 with an expected error of 2 and 4. Taxonomic classification was performed against the SILVA v138 database [63].

141 ASVs detected in non-template and extraction controls were considered as contaminants and removed using *decontam* v1.22 [64], as well as ASVs identified as unassigned (341), eukaryotic (4), chloroplast (185), mitochondrial (280) and singletons (30). A total of 8,836,875 high-quality reads with a mean sequencing depth of 53,234.19 ± 32,574.94 per sample were obtained from the 16S rRNA amplicon sequencing approach. These reads represented 16,545 ASVs from 41 phyla, 330 families and 913 genera. 10,319 ASVs were assigned to samples from adult fish, while 9102 ASVs were detected in samples from juvenile fish. Appropriate sequencing depth was estimated by the construction of a rarefaction curve (see Additional File 2, Figure S1) with *vegan* v2.6.4 [65]. Three samples, which did not reach sufficient sampling coverage (less than 1000 reads per sample), were removed from the dataset for the subsequent analysis. Cumulative sum scaling (CSS) normalization was then applied to the ASV table as it effectively mitigates biases due to varying sequencing depths and compositional constraints in highly sparse datasets.

### Statistical analysis

A preliminary permutational multivariate analysis of variance (PERMANOVA) based on Bray–Curtis dissimilarity indicated that age significantly contributed to the observed differences between samples. Consequently, the dataset was analyzed separately for each age group as the strong influence of age might otherwise mask treatment effects (see result section for further explanation). The effects of weight, size, external water parameters and sex were initially tested and showed no significant impact (p-value > 0.05; data not shown); thus, these variables were omitted from any subsequent analyses. Exploratory analysis furthermore identified 19 samples as outliers, as these samples showed extremely low ASV Richness with dominance of one or two ASVs accounting for around 95% of sequences. Consequently, these samples were not included into the downstream analyses.

Unless specified otherwise, analyses were conducted with the R packages *phyloseq* v1.46 [66] and *microbiome* v1.24 [67]. Plots were prepared with *ggplot2* v3.3.4 [68] and *ggpubr* v0.6 [69].

Microbial alpha diversity was estimated at ASV level between sample groups based on Observed ASV Richness. Prior to statistical analysis, data distribution and homoscedasticity were checked with *Levene* test (R package *car* v3.1.2; [70]) and visually via Q-Q plots. To investigate the effect of age, treatment, and sampling timepoint on microbial diversity, a generalized linear model implemented in the *lme4* [71] package was applied. Optimal model fit was evaluated prior based on the goodness-of-fit criteria using the *stepAIC* function of the package *MASS* [72]*.* Differences in microbial community composition between control and treatment groups over the experimental period were determined based on Bray–Curtis dissimilarities by *pairwiseAdonis* v0.4.1 [73] using 10.000 permutations and Bonferroni as FDR correction. Clustering patterns between samples were visualized using non-metric multidimensional scaling (NMDS). To assess the homogeneity of group dispersions and validate the robustness of the PERMANOVA results, the *betadisper* function from the *vegan* R package [65] was employed.

The core microbiome per treatment group was defined as ASVs occurring in 70% of samples with a minimum detection threshold of 0.0001%. For the identification of differentially abundant ASVs between groups receiving either antibiotic and/or antiparasitic treatment and un-exposed control samples, DESeq2 was applied [74]. DESeq2 employs a negative binominal generalized linear model approach to detect differences in ASV counts between groups. Due to the complexity of the model including four treatment and five timepoint levels, likelihood ratio test was used, p-values were adjusted by Benjamini–Hochberg method. ASVs were considered as enriched or depleted with a log2-fold change > 2 and an FDR-adjusted p-value < 0.05.

Microbial co-occurrence networks were inferred using SPIEC-EASI (spare inverse covariance estimation for ecological associations; [75] implemented in the R package *NetCoMi* v1.1 [76]. SPIEC-EASI is particularly suitable for microbiome data due to its ability to handle sparse and compositional data, providing accurate and stable network inferences. As multivariate statistics did not indicate any significant differences at t28 between the different treatments and control, all treatment samples at t28 were pooled into one cohesive network per age. Separate networks were then constructed for each control group and post-treatment group, including only ASVs occurring in at least three samples. Data was normalized via the default clr-transformation to address compositional bias. Highly interconnected modules of nodes within the networks were identified using the *cluster_fast_greedy* algorithm. Highly influential hub ASVs were identified with the R packages *influential* v2.2.9 [77] and igraph v2.0.3 [78], applying the Integrated Value of Influence (IVI) algorithm. This method combines multiple network topological properties and centrality metrics (e.g., betweenness, degree, h-index) to robustly classify key hubs. ASVs with an IVI score above 50 were considered as hub nodes. Furthermore, the taxonomic composition of hub ASVs on genus level was plotted using *complexHeatmap* v2.18 R package [79].

## Results

### Age shapes the bacterial composition in the gastrointestinal tract of brown trout not exposed to antibiotics

**Fig. 1:**
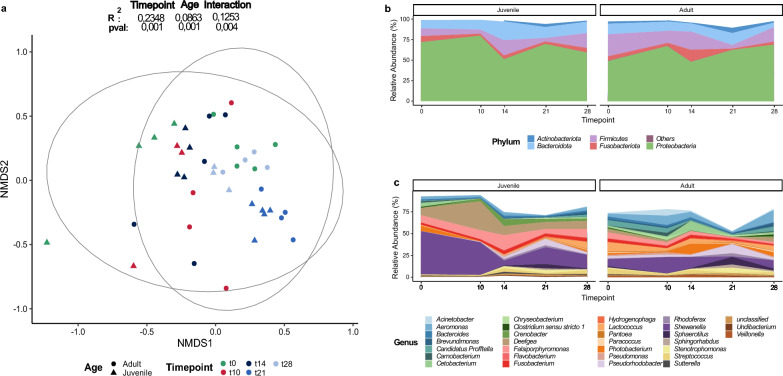
Effect of trout age on gut microbiome composition. **a)** NMDS plot representing Bray Curtis dissimilarities between control samples from adult (dot) and juvenile (triangle) fish over 28 days, comparing compositional differences between age groups independently of treatment. (stress = 0.1929). PERMANOVA results are included. **b)** Temporal dynamics of bacterial phyla present in control samples from juvenile and adult fish. Phyla with < 1% rel. abundance are grouped as “Others”. **c**) Temporal dynamics of top 30 bacterial genera for each control group

Throughout the experiment, neither fish length nor weight differed significantly between the treatment groups (see Supplementary Table S1, Additional File 1). Additionally, no mortalities or clinical signs of illness were observed in any group, indicating that antimicrobial treatment did not affect overall fish health. We initially focused on characterizing the diversity and taxonomic profile of the unaltered digesta-associated microflora in juvenile and adult fish under controlled conditions throughout the experimental period. Observed ASV richness remained stable over time and was not significantly correlated with host age (see Additional File 1, Table S4; Additional File 2, Figure S2). As shown in Fig. 1a, non-metric multidimensional scaling (NMDS) based on Bray–Curtis dissimilarities revealed distinct clustering by host age (two-way PERMANOVA; R^2^ = 0.086, p_adj_ = 0.001) and sampling timepoint (R^2^ = 0.235, p_adj_ = 0.001). Despite stable conditions within the tanks and the absence of external stressors like antibiotics, the microbial communities exhibited significant temporal oscillations over 28 days, accounting for 23.5% of observed variation independently of host age. The microbiome composition was mainly represented by five phyla, with *Proteobacteria*, *Bacteroidota* and *Firmicutes* being predominant (see Fig. 1b). Consistent with the PERMANOVA results, significant differences at genus level were observed between age groups. Adult fish predominately harbored ASVs classified as *Shewanella, Aeromonas, Photobacterium, Lactococcus* and *Cetobacterium*. *Shewanella*, *Aeromonas* and *Fusobacterium* were consistently present across all timepoints in both groups as core taxa, whereas the abundance of the other genera was notably reduced in juvenile fish (Wilcoxon, p < 0.05). Conversely, *Deefgea*, *Falsiporphyromonas* and *Crenobacter* were highly dominant in juveniles (Fig. 1c; Wilcoxon, p < 0.05).

In addition to the dominant genera, low-abundant but highly prevalent ASVs assigned to *Bacteroides* and *Streptococcus* were detected in the core microbiome analysis (see Supplementary Figure S3, Additional File 2).

Finally, comparison of bacterial co-occurrence networks for each control group revealed distinct differences in the overall network structure and interplay within these communities.

Co-occurrence networks in the gut microbiome of juvenile trout were less interconnected compared to adults, with reduced information flow between nodes, as reflected by fewer edges (3142 vs. 6135), lower average degree (25.76 vs. 38.30) and betweenness centrality (517.56 vs. 553.32), alongside a slight increase in path length. The higher proportion of positive edges suggests a more cooperative microbial community, where key taxa mediate critical interactions within a simplified network structure (see Supplementary Table S5, Additional File 1). Moreover, the taxonomic composition of hub ASVs identified based on IVI measure differed significantly between adult and juvenile networks. The adult network harbored a much higher number of influential nodes, including *Brevundimonas*, *Aeromonas, Cetobacterium, Clostridium sensu stricto 1* and *Carnobacterium*. In contrast, the juvenile network had a limited number of low-abundant hubs, such* as Fusobacterium, Sphingomonas* and *Pseudomonas* (see Supplementary Figure S4, Additional File 2). These findings support the notion that the established gut microbiome forms a more densely connected network with functionally important ASVs occupying central roles, potentially contributing to increased stability and resilience to external perturbations.

### Differential responses of the gut microbiome to florfenicol and peracetic acid treatment are mediated by trout age

**Fig. 2:**
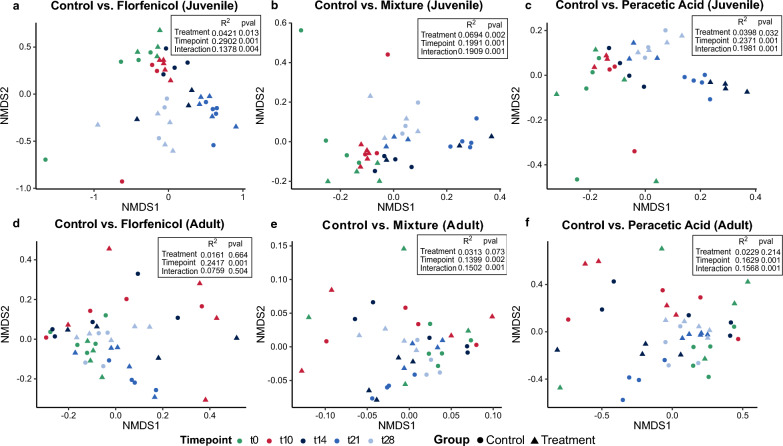
Effect of treatment on bacterial ß-diversity. NMDS ordination plots of beta diversity in gut communities from juvenile (**a–c**) and adult brown trouts (**d–f**) based on Bray–Curtis distance comparing the respective control groups with each treatment. Colors represent the different sampling timepoints (t0 = pre-treatment, t10 = treatment, t14–t28 = post-treatment), while shape indicates to which group each sample belongs. NMDS stress values < 0.2 indicated that ordinations were sufficiently robust, providing a reliable representation of the original data. Pairwise PERMANOVA results for each comparison are indicated on each plot

All treatments induced pronounced shifts in the composition of bacterial communities in the gut of juvenile brown trout, displaying similar temporal oscillations across different sampling timepoints as observed in the control groups (see Fig. 2a–c for detailed two-way PERMANOVA results). As shown in the NMDS ordination plots, the strongest separation between community dissimilarities was observed in the early post-treatment phase (4 days up to 11 days after the last administration of drugs), while the distinction was less clear during the treatment phase, particularly in the mixture and PAA groups. However, only 4.21%, 6.94% and 3.98% of the variability in community composition were explained by florfenicol, mixed and peracetic acid treatment. In contrast, sampling timepoint explained 29.02%, 19.91% and 23.71% of total variance, suggesting that temporal dynamics exerted a greater influence on overall microbiome composition than antibiotic exposure. Furthermore, the relatively low R^2^ values suggest that other unmeasured environmental or biological factors may play a significant role in shaping the microbiome response to external stressors like antibiotics and antiparasitics.

Notably, similarly strong treatment effects were absent in adult fish. As illustrated in Fig. 2d–f, clustering patterns among samples of treated and untreated adult fish were primarily driven by sampling timepoint regardless of treatment (p_adj_ = 0.001 for all comparisons). A significant interaction effect between treatment and sampling timepoint for both adult and juvenile fish indicated that the impact of treatment on the microbial community might not be uniform throughout the post-exposure phase.

Consequently, data were analyzed separately by sampling timepoint to identify microbial key responders driving observed differences in the following. However, the observed changes in microbiome composition did not correspond to a reduction of ASV richness or evenness, although a slight decrease was noticeable during the exposure phase in all treatments in adult trout (see Supplementary Figure S2, Additional File 2). Despite clear clustering patterns shown in the ordination plots between treated and control communities, there was a lack of statistically significant differences in the alpha diversity of the gut microbiota of juvenile trout.

### Microbial key responders in the gut microbiome of brown trout to florfenicol and peracetic acid

**Fig. 3:**
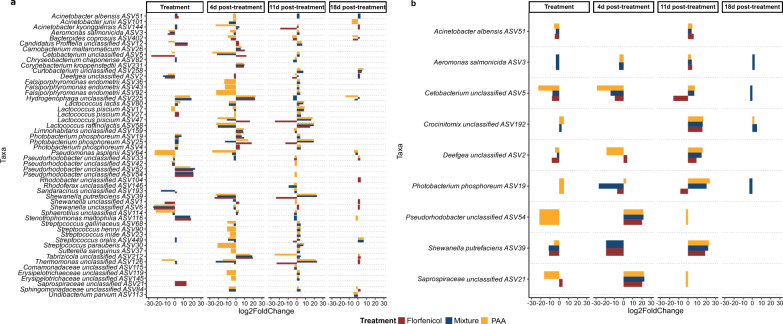
Microbial key responders. Barplots representing log2fold change of differentially abundant ASVs between control and treatment groups of **a** juvenile and **b** adult brown trout. Only ASVs with a log2fold change over 2 (increased abundance) oder—2 (decreased abundance) and p_adj_ < 0.05 were considered

Out of 9102 ASVs detected in juvenile fish, 52 ASVs showed significant differential abundances in the treatment groups compared to the control group, whereas only 9 out of 10,319 ASVs differed across all treatments in adult fish. Figure 3a (juvenile) and 3b (adult) illustrate the log2foldchange of ASVs with altered abundance patterns during treatment in the early and late post-treatment phases. As expected from the beta diversity analysis, taxa exhibited similarly strong temporal dynamics over the duration of the experiment. The most pronounced changes were observed between 4 to 11 days post-treatment, with most changes stabilizing after 18 days of withdrawal. Taxonomic shifts observed in bacterial communities from juvenile trout during the exposure phase to either florfenicol, peracetic acid, or combined treatment were largely driven by ASVs assigned to *Aeromonas salmonicida* (ASV3), *Deefgea sp*. (ASV2), *Shewanella sp.* (ASV6) and *Pseudomonas asplenii* (ASV64) among others, which significantly decreased in read abundance between day 0 (pre-treatment) and day 10 (last day of treatment) compared to untreated fish. In addition, PAA treatment reduced the abundance *Lactococcus piscium* (ASV17), although these changes were not detected in the other treatment groups. Interestingly, two ASVs equally classified as *Lactococcus piscium* (ASV27, ASV47) either showed no immediate response or even displayed increased read abundance in the case of florfenicol exposure. Moreover, both florfenicol and PAA treatment strongly diminished the abundance of *Cetobacterium sp.* (ASV5) until 4 days post-treatment. These shifts were accompanied by an enrichment of ASVs classified as *Photobacterium phosphoreum* (ASV19, ASV25, ASV4) and *Hydrogenophaga sp*. (ASV225), which persisted after 4 up to 11 days of antibiotic withdrawal. Two potential opportunistic pathogens, *Stenotrophomonas maltophila* (ASV116) and *Streptococcus oralis* (ASV449), were also notably enriched. Enrichment of these ASVs was primarily mediated by florfenicol or the mixed treatment, whereas PAA tended to trigger an overall decrease in read abundances. Post-treatment dynamics were predominately driven by singular PAA application or combined treatment with florfenicol and PAA, rather than by florfenicol alone. Although unaffected during the treatment phase, the following ASVs among others were drastically depleted following a 4-day withdrawal of PAA: *Falsiporphyromonas endometrii* (ASV43, ASV36, ASV92), *Streptococcus henryi/gallinaceus/iniae/parauberis* (ASV68, ASV90, ASV23, ASV449, ASV30), *Shewanella putrefaciens* (ASV39). Additionally, *Lactococcus raffinolactis* (ASV58) and *Lactococcus piscium* (ASV47) showed significantly reduced abundance in the mixed and PAA-treated groups. *Bacteroides coprosuis* (ASV402), which started to decline within florfenicol and PAA-treated fish (but not the combined treatment), remained notably depleted in the gut of juvenile fish up to 18 days post-treatment.

However, by day 21 (11 days post-treatment), we observed that most of these effects were strongly reversed, with previously depleted ASVs, especially by PAA and mixed treatment, showing significantly elevated abundances compared to untreated fish. Among these were potential opportunistic pathogens including *Streptococcus sp.* and *Aeromonas salmonicida*.

In addition to the depletion of important gut commensals like *Bacteroides coprosuis* up to 18 days post-treatment, the later stages of the withdrawal phase showed continuous depletion of *Hydrogenophaga sp.* and *Pseudomonas asplenii* despite their initial increase. Persistent elevated abundances were detected for several other taxa, including *Shewanella sp.*.

We found considerable overlap between differentially abundant ASVs in the intestinal bacterial consortia of adult and juvenile fish, following similar temporal trends throughout the 18-day withdrawal phase. Unlike juvenile fish, the dominant taxa in the gut microbiome of healthy adult trout were largely unaffected, except for *Cetobacterium sp.* (ASV5) and *Aeromonas salmonicida* (ASV3).

### Alterations of microbial co-occurrence pattern after treatment cessation

**Fig. 4:**
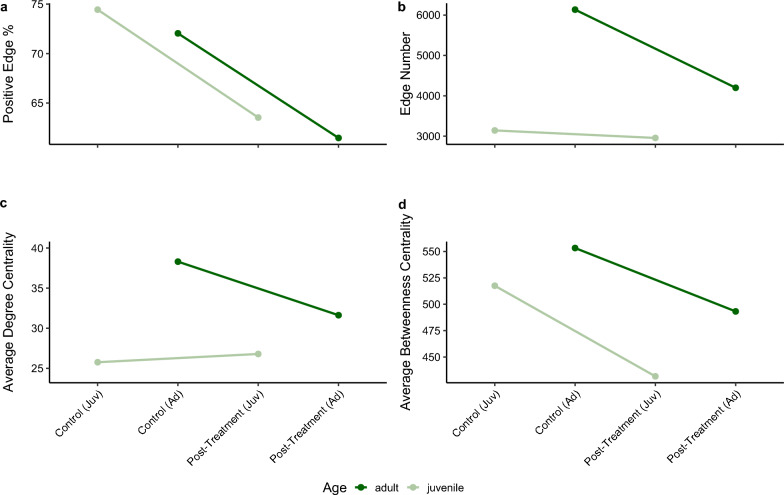
Changes in network properties between control and post-treatment networks. Plot shows trends in four network metrics estimated with NetCoMi and igraph: **a**) positive edge percentage, **b)** number of edges, **c**) average degree centrality and **d)** average betweenness centrality. Lines representing adult samples are colored dark green, juvenile samples are colored in light green

According to pairwise multivariate analyses by timepoint, microbial communities largely recovered their pre-exposure abundance and composition levels 18 days after treatment cessation, with no significant differences between the treatment groups and controls. However, lasting effects of antibiotic disruption are not only reflected in changes in bacterial diversity but also in how microbes interact within the community. Shifts in microbe-microbe-interactions can significantly affect the stability and function of the microbial consortium, revealing more subtle disruptions that may persist despite the recovery in overall abundance. To explore this question, we examined whether network topology and centrality measures of microbial key responders in post-treatment networks returned to their pre-exposure state. For this, we combined all treatment samples from the last sampling timepoint (t28) into one cohesive network per age group. Both post-treatment networks remained in a more competitive state, as indicated by a significant reduction of positive interactions (74% to 63% and 72% to 61%). As shown in Fig. 4, this was also reflected in decreased node connectivity and loss of connector species.

**Fig. 5:**
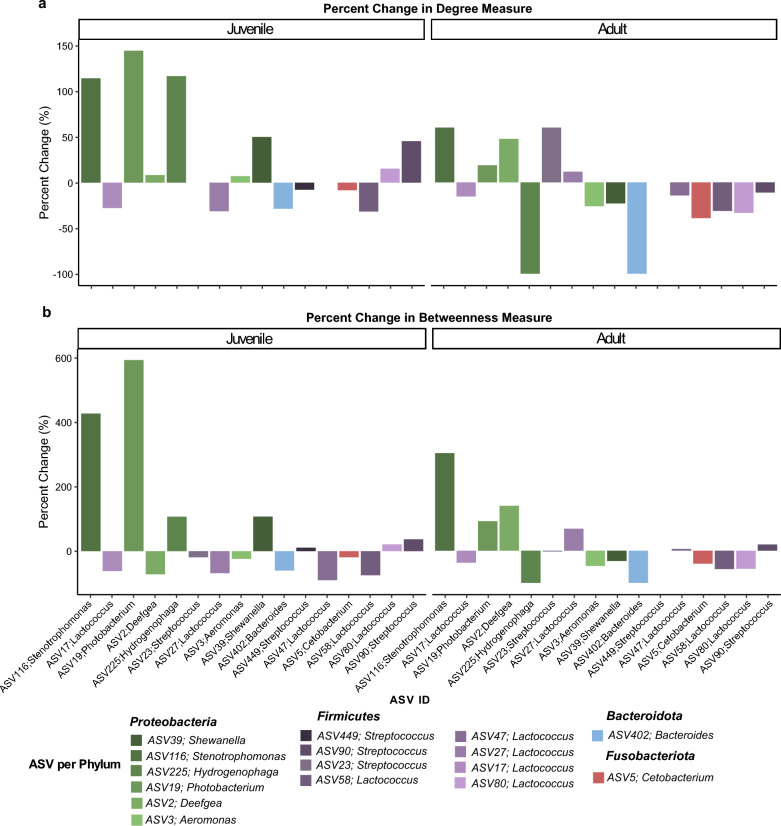
Relative changes of network positions of microbial key responder ASVs identified by DESeq2. The plot shows the percentage change in degree (**a**) and betweenness centrality (**b**) for juvenile and adult brown trout samples. Each bar represents an individual ASV, with bars colored according to the phylum to which the ASV belongs. Missing bars indicate that the metric did not change

Although the abundance of microbial key responders identified by DESeq2 largely returned to control levels, their positions within the network remained altered. Notably, the degree connectivity of three ASVs from potentially pathogenic genera (*Stenotrophomonas, Shewanella* and *Streptococcus*) increased by 114% relative to the control in juvenile trout (see Fig. 5). More importantly, their betweenness centrality surged up to 424%, signifying that these potentially harmful taxa may take more influential roles within the post-treatment state. This shift suggests that pathogens may exploit ecological disturbances caused by antibiotic treatment to occupy central positions, possibly gaining a competitive advantage by controlling key microbial interactions. In contrast, similar pronounced changes in centrality metrices were largely absent in adult trout, except for *Stenotrophomonas* and *Photobacterium*. Despite this, highly prevalent genera such as *Lactococcus*, *Bacteroides*, and *Cetobacterium* in both age groups lost their central positions within the network, even though their abundances had recovered. This was indicated by a drastic relative decrease of individual degree and betweenness measures by up to 100%. This decoupling of abundance from network influence may indicate a diminished functional role as connectors, which could reduce the overall resilience and functional stability of the community.

## Discussion

The growing demand for affordable animal protein has led to a rapid expansion of aquaculture production, driving increased reliance on antibiotics. This practice raises concerns, as broad-spectrum antibiotics can disrupt host-associated microbiomes and negatively affect host physiology. Recent evidence suggests that the microbiome’s response to antibiotic exposure is not uniform but varies depending on co-variables such as age and health status and the specific drugs used [44, 48]. To our best knowledge, this study is the first to investigate the effects of PAA on the gut microbiota of farmed fish. Contrary to our initial hypothesis, we found that even low concentrations (0.005%) of PAA were sufficient to induce significant and lasting alterations to the gut microbiome composition and interaction patterns, exceeding the effects of florfenicol. This challenges existing assumptions about PAA’s minimal toxicity. Additionally, we confirmed our hypothesis that fish age is a key determinant in shaping microbial community dynamics, particularly under external stress.

### Microbiome maturation drives community resilience in adult brown trout

In our study, fish age drastically affected the gut microbiome composition regardless of treatment, although no differences in ASV richness between juvenile and adult fish was detected. These results align with findings from recent studies, which have shown major compositional shifts across development stages in different fish species, even when fish were in a relative late stage of development and already exhibited physiological and nutritional characteristics akin to mature fish. The observed proportions of dominant phyla, namely *Proteobacteria, Bacteroidota*, *Firmicutes* and *Fusobacteria*, were found to be consistent with previously reported data for mature gut microbiomes of various freshwater species [11–15]. Among them*,* ASVs representing *Cetobacterium sp.* were strongly associated with adult fish. The genus *Cetobacterium* contains two vitamin B12 producing species, *C. ceti* and *C. somerae*, which are frequently identified as core taxa in a diverse range of (carnivorous) freshwater fish [9, 80–82]. Although vitamin B12 (cobalamin) is an essential co-factor for maintaining the functionality of the nervous system, formation of red blood cells, DNA synthesis and necessary for key microbial metabolic processes, genes encoding for anaerobic cobalamin synthesis are only present in around 25% of bacterial and archaeal clades [83]. A recent study by Qi et al. [84] emphasized the significance of *C. somerae* for host physiology in zebrafish through conferring colonization resistance against infections with *Aeromonas hydrophila* and modulating the equilibrium of healthy gut communities through promotion of host-microbe and microbe-microbe-interactions, thereby enhancing overall stability. Furthermore, probiotic supplementation of vitamin B12 increases intestinal levels of acetate, propionate and butyrate, essential short-chain-fatty acids (SCFAs) involved in gut barrier integrity [85]. *Firmicutes* are highly prevalent in the autochthonous gut microbiota of fish and mammals and several genera within this phylum are known to contribute to the host’s nutrition through SCFA production and degradation of dietary fiber [86, 87]. Similar to previous results, *Lactococcus sp.* and *Streptococcus sp*. constituted a substantial fraction of the core microbiome of adult and in the case of *Streptococcus* also juvenile brown trout [12, 88–90]). Several prevalent ASVs in both age groups identified in this study represent zoonotic pathogens, including *S. parauberis* [91], *S. iniae* [92, 93] and *L. garviae* [94]*,* which cause significant economic losses in fish farms through high mortality rates [95]. Several *Lactococcus* species (*L. lactis, L. piscium, L. raffinolactis*), have been investigated for their potential as probiotic feed supplements in gilthead sea bream [96], zebrafish [97] and common carp [98]. These species exhibit antagonistic activity against invading pathogens through production of bacteriocins and pH-reducing lactic acid. By producing antimicrobial substances and potentially engaging in negative biotic interactions, these *Lactococcus* species may inhibit the overgrowth of opportunistic pathogens, thereby contributing to a more stable and resilient microbial community [99]. Despite the limited understanding of the functional roles and interaction patterns of *Cetobacterium* and *Lactococcus*, their high prevalence across various fish species suggests they play significant roles in their respective ecological niches and may act as keystone taxa, contributing to the stability and functional plasticity of mature fish gut microbiomes. Indeed, network analysis identified *Cetobacterium sp.* as a highly influential hub, suggesting it plays a vital role in maintaining community stability by mediating critical interactions and facilitating metabolite exchange within the network [100]. Additionally, the current results support the hypothesis that the bacterial community selectively favors beneficial taxa during development, as evidenced by their consistent presence as core microbes and their low abundance in juvenile brown trout. *Proteobacteria* were consistently present across all age groups and sampling timepoints, including heterogenous genera such as *Aeromonas*, *Photobacterium* and *Shewanella*. Although they are commonly found as symbionts and are involved in the production of digestive enzymes (chitinases, lipases, proteases etc.), all these genera contain highly relevant pathogens for trout aquaculture [101–103]. Due to substantial inter-individual variation and the strong influence of species and developmental stage, direct comparisons with other studies on juvenile fish are limited. *Deefgea* and *Falsiporphyromonas*, which were indicative for the juvenile stage, have been mentioned in some other studies [30, 34], but knowledge on their specific function in the gastrointestinal tract or aquatic ecosystems on a broader scale is incomplete. Our study extends existing data by demonstrating that the gut microbiome of juvenile trout shows increased volatility in comparison to adults, based on a network approach. The capacity of microbial communities to withstand external stressors is determined by tight interactions and efficient resource distribution within a complex, stable network of interconnected metabolic processes. The stability of the host-associated microbiome is of critical importance for the maintenance of beneficial symbionts involved in nutrient utilization, immune development, and colonization resistance, which are essential for the long-term health of the host [104, 105]. Conversely, substantial alterations in microbial composition and interaction patterns are frequently indicative of dysbiosis and impaired health. In adult fish, the microbiome forms a more interlinked and less modular network, promoting efficient communication and nutrient exchange between nodes. This structure likely enhances resilience by evolving towards fewer but more specialized connections, reflecting the metabolic needs of adult fish, and increases functional redundancy. As a result, the adult microbiome is better equipped to minimize localized failures, with other members compensating for the loss of certain species, maintaining community equilibrium, and enhancing colonization resistance.

### Age-dependent microbiome responses to florfenicol and peracetic acid treatment

The observed differences in community composition and network architecture between juvenile and adult fish may help explain the strongly age-dependent impact of florfenicol and peracetic acid on the gastrointestinal microbiota of brown trout. In juveniles, shifts in the bacterial community were evident immediately in response to all three treatments, which did not recover up until 11 days post-treatment. Given previous results from studies on florfenicol use in aquaculture, we anticipated a moderate effect on the gut microbiome of adult fish, albeit to a lesser extent than in juvenile fish. Changes in beta diversity along with an increase in *Proteobacteria:Bacteroidota* and *Proteobacteria:Firmicutes* ratios have been consistently reported [29]. However, minor shifts were observed in adult fish. Although this may indicate that the more interconnected and resilient mature microbiome is better equipped to withstand certain disturbances, it cannot be excluded that the minimal effects observed may be partly related to improper feed consumption and florfenicol metabolism. While the rearing conditions were optimal for the needs of brown trout, low water temperatures (9–11 °C) may have altered pharmacokinetics of florfenicol, reducing florfenicol metabolism in some fish [106]. Additionally, differences between gut regions could explain the lack of significant effects, as antibiotic impact varies across sections [32, 42]. The observed temporal dynamics, regardless of treatment, suggest inherent microbial community variability, with high inter-individual variation potentially masking significant differences between groups.

To the best of our knowledge, this study is the first to demonstrate that external PAA application led to significant alterations in the gut microbial communities of brown trout, exceeding those of florfenicol. While no significant changes were detected during the treatment phase, notable shifts in microbial communities emerged between days 4 and 11 post-treatment. While the low concentrations of PAA used in this study may exert a bacteriostatic rather than bactericidal effect, the delayed microbial response observed was unexpected. Previous studies on PAA application in RAS have shown minimal disruption to microbial communities in rearing water and biofilters during pulse treatments [39, 107], with some studies even reporting increased bacterial counts [40]. These findings suggest that while PAA can effectively target specific strains, its broader impact on microbial communities, particularly in complex environments like the gut, is more nuanced and context dependent. The delayed response observed could be due to several underlying mechanisms. One possibility is that the gut microbiome initially resists PAA, possibly due to prior exposure. The alterations post-treatment may reflect a delayed restructuring of the community, likely due to the gradual depletion of key species or changes in gut conditions that favor microbes capable of degrading PAA residues.

Notably, *Hydrogenophaga*—a common methylotrophic denitrifier found in activated sludge [108], wastewater [109], and amphibians [110]—significantly increased in abundance following PAA exposure. Several *Hydrogenophaga* strains are known for their ability to oxidize hydrogen and arsenite [111], as well as degrade xenobiotic pollutants like xylene [112] and triclosan [30]. In this study, *Hydrogenophaga* was initially low in abundance, but its increase suggests a selective advantage, potentially due to its resistance to hydrogen peroxide (a byproduct of Wofasteril 400©) and its ability to utilize fish-derived nitrogenous waste. Although we did not measure nitrogen species beyond ammonium or quantify denitrification rates through qPCR, it is possible that the elevated presence of *Hydrogenophaga* may contribute to maintaining balanced nitrogen levels in the gut and in the water, by degrading toxic nitrogenous compounds that might otherwise harm the host. However, this interpretation is based on the known metabolic traits of this genus and its involvement in nitrogen cycling through denitrification in other systems [111, 113], but remains speculative in the context of this study.

Florfenicol and PAA treatments led to the depletion of key commensals like *Cetobacterium*, *Lactococcus*, *Bacteroides* and *Pseudomonas*. Given the importance of *Cetobacterium* and *Lactococcus* in nutrient cycling and gut homeostasis in adult fish, depletion of these taxa during ontogeny may result in dysbiosis, negatively affecting the growth and health of farmed fish. If these commensals are slow to recover or fail to recover at all, essential functions may be lost, hereby challenging efforts to maximize aquaculture production to meet the growing global demand for affordable animal protein.

Our study reveals successional trends post-treatment, where opportunistic pathogens like *Aeromonas*, *Shewanella*, *Stenotrophomonas, Streptococcus* and *Acinetobacter* initially declined but later surged, sometimes exceeding their initial abundance. Increased prevalence of opportunistic (fish) pathogens is a common denominator of antibiotic-induced dysbiosis [30, 31, 34]. Due to its high genetic plasticity and elevated horizontal gene transfer rates, *Aeromonas* serves as an excellent proxy for the acquisition and spread of ARGs under selective pressure, making it a suitable indicator species for AMR surveillance in aquatic ecosystems [103, 114]. High multidrug resistance levels observed in subpopulations of these species, combined with their R-strategy traits, likely explain their ability to outcompete other taxa, including beneficial microbes, in the later stages of antibiotic cessation, effectively occupying vacated ecological niches [115, 116]. In the current study, fish showed no obvious signs of disease, but the temporary increase in pathogen load may lead to a higher risk of infection, especially under stressful rearing conditions or suboptimal water quality. In a stable system, commensals suppress overgrowth of facultative pathogens, which are usually part of the normal flora, through negative feedback loops. Changes in the ratio of pathogens to commensals may foster an elevated risk of disease within the population. The rise in opportunistic pathogens poses risks not only to fish health but also to human health. The dissemination of ARGs from aquatic environments, through handling or consummation of contaminated fish or contact with rearing water, might contribute to the global AMR crisis. Furthermore, some of the pathogens identified, including *Streptococcus* and *Stenotrophomonas,* are rare causative agents of septicemia in humans [117, 118]. Contrary to previous reports that described synergistic effects of antibiotics and biocides, such as enhanced antimicrobial efficacy and increased rates of horizontal gene transfer (HGT) and ARGs [119, 120], we did not observe an amplified effect in the combined treatment group of florfenicol and peracetic acid, at least not at the taxonomic level. While synergistic effects have been documented against clinical pathogens like *Staphylococcus aureus* [121], the dynamics within a complex system like the gut microbiome of brown trout may differ. It is also possible that, while we did not detect synergistic effects on the taxonomic composition, there may have been changes in the resistome, that were not captured by our approach. Previous studies have noted that sub-inhibitory concentrations of biocides can stimulate ARG transfer, but without direct assessment of ARG, we cannot confirm if this mechanism played a role.

### Microbial co-occurrence network reconfiguration following treatment and recovery

Although the data suggests that microbial communities recover from antimicrobial treatment when not exposed to further antibiotics or antiparasitics, network analysis showed that the interaction patterns remain significantly altered. Post-treatment samples of both ages displayed reduced connectivity compared to control groups, transitioning into a more competitive state. This shift implies that exposed microbiomes may act in a more self-sufficient and antagonistic manner. Competitive communities, as described by Machado et al. [122], tend to retain versatile metabolic capabilities, enabling them to exploit available nutrients and outcompete other species for open niches. Instead of mutualism, microbes prioritize survival under stressful conditions through resource acquisition and competition. This behavior favors R-strategists and species with high genetic plasticity, which are often more resilient to antibiotic reduction. Our data supports this hypothesis in two ways: a) reduced betweenness and connectivity indicating fewer interactions and mutual dependencies and b) certain opportunistic pathogens like *Shewanella* and *Stenotrophomonas* gained more central roles within the post-treatment networks relative to the controls, indicating an increase in their influence on the community. This was particularly evident in juvenile trout. While competitive communities generally exhibit a higher resilience to antibiotics, this comes at a cost. Cooperative interactions are typically supported in host-associated systems, as they provide support in pathogen suppression, nutrient cycling, and gut barrier integrity. Mutualistic species often engage in cross-feeding, breakdown of polysaccharides and SCFA production, which maintain gut health [123]. A reduction in these interactions may leave the host more susceptible to pathogen invasion and metabolic imbalances, especially during the vulnerable juvenile stage. Despite regaining their initial abundance, several commensals (*Lactococcus*, *Cetobacterium*) occupy less central position with the post-treatment networks. This suggests that recovery at the taxonomic level may not equate to functional restoration. To fully evaluate this question, long-read metagenome sequencing combined with metabolomics should be employed to accurately infer changes in the functionality of microbial responders and the overall consortium. Additionally, the use of long-read sequencing techniques enables precise identification of microbial taxa at the species or even subspecies level, which is particularly critical for pathogen detection. This method also allows for better correlation with antibiotic resistance patterns and the potential for co-selection of ARGs, providing a more comprehensive understanding of the impacts of combined antibiotic treatments. Aquaculture is already recognized as a significant reservoir for AMR, with implications not only for fish health but also for the broader One Health perspective, where the spread of resistance can affect human and animal health globally.

While this study provides valuable insights into the age-dependent effects of florfenicol and peracetic acid on the gut microbiota of brown trout, some methodological limitations should be considered when interpreting the findings. The use of fecal material, widely accepted in fish gut microbiome research, has been shown to capture a representative fraction of the intestinal microbiota [50–52]. However, it does not fully account for mucosa-associated bacteria, which form distinct microbial niches along the gastrointestinal tract. These mucosal communities play critical roles in host-microbe interactions and may respond differently to antimicrobial treatments. As a result, certain treatment effects specific to mucosal niches could not be fully captured by the sampling approach used in this study.

Additionally, microbial composition and activity vary significantly across gut regions. By relying on fecal matter from the distal section of the gut, localized effects of antimicrobial treatments, such as those reported by Kokou et al. [42], may have been masked. This limitation is particularly relevant for interpreting data from adult fish, whose microbiota exhibit more specialized and regionally distinct profiles compared to the more homogenized gut communities of maturing fish. This distinction may explain why treatment effects in adult fish were less apparent in this study.

Lastly, the relatively small sample size, though comparable to other fish microbiome studies, presents another limitation. High inter-individual variability, although well-documented for fish [124, 125], may have obscured significant treatment effects. Larger sample sizes in future studies could not only improve the detection of statistically significant differences but also enhance the robustness of microbial co-occurrence network analyses. This is particularly important for evaluating stability metrics, which typically require higher statistical power to yield robust results [126]. Despite these constraints, the network-based approaches employed in this study remain a powerful tool for understanding how microbial interactions are disrupted by antimicrobial treatments, extending beyond abundance-based metrics to reveal community-level dynamics. Future studies incorporating larger sample sizes, region-specific sampling, and functional analyses will further improve the ability to elucidate the complex responses of fish gut microbiomes to antimicrobial exposure and aid in defining evidence-based measures to optimize husbandry practices in aquaculture.

## Conclusion

Maintaining a stable and functional microbiome is essential for preserving fish health and resilience, which in turn is critical for ensuring optimal production efficacy in aquaculture. The strong age-dependent patterns observed in this study emphasize the importance of responsible antibiotic stewardship and evidence-based policymaking, particularly in hatcheries where juvenile fish are most vulnerable. Our results indicate that juvenile trout experience more pronounced and long-lasting shifts in microbiome composition following florfenicol and PAA treatments, making them more susceptible to dysbiosis. These shifts were characterized by an increase in opportunistic pathogens like *Aeromonas* and a decline of key beneficial taxa. Similar treatment effects were absent in adult trout. The use of peracetic acid as an antiparasitic and disinfectant should be carefully re-evaluated, as its use is associated with significant risks of microbiome disruption, particularly in younger fish. Repeated exposure, such as through disinfected tank equipment or water treatment, may exacerbate these disruptions and warrants careful evaluation in practice. More studies are necessary to fully decipher the exact impact of PAA on the fish gut, spanning different dosages, fish species, and rearing systems. Such knowledge is not only crucial in improving animal welfare in aquaculture for human consumption, but also for supportive breeding programs where performance of hatchery-reared fish stocked in the wild is particularly crucial.

To mitigate adverse effects of antimicrobial treatments, alternative strategies should be explored in aquaculture. These may include the improvement of husbandry practices that reduce reliance on antibiotics and biocides, and the use of probiotics like *Lactobacillus* and *Pediococcus* [99] to enhance microbiome resilience in juvenile trout. Additionally, treatment protocols in hatcheries should be carefully reconsidered to minimize early-life microbiome disturbances, focusing on enhancing natural microbial defenses rather than relying on repeated chemical interventions.

## Supplementary Information

Additional file 1.

Additional file 2.

## Data Availability

Raw sequence reads from demultiplexed samples analyzed in this study have been deposited in the NCBI Sequence Read Archive (SRA) under BioProject accession PRJNA1162705. R Scripts used for data analysis of this dataset and the dataset analysed during the current study (phyloseq object containing the unnormalized ASV table and respective metadata) are available in GitHub at https://github.com/lstreb713/TroutGut.

## Abbreviations

RAS: Recirculating aquaculture system
ARG: Antibiotic resistance gene
PAA: Peracetic acid
MEG: Mobile genetic element
NMDS: Non-metric multidimensional scaling
IVI: Integrated value of influence
