# Supplementary material for: Age matters: exploring differential effects of antimicrobial treatment on gut microbiota of adult and juvenile brown trout (Salmo trutta)
Source: Anim Microbiome. 2025 Mar 16;7:28. doi: 10.1186/s42523-025-00391-2 (PMC11910850; doi:10.1186/s42523-025-00391-2)
Supplement: Supplementary file 1 — Additional file 1. [file 42523_2025_391_MOESM1_ESM.docx]

**Additional File 1 of “Age Matters: Exploring differential effects of antimicrobial treatment on gut microbiota of adult and juvenile brown trout (S*almo trutta fario*)”, containing all supplementary tables:**

**Table S1:** Size and weight distribution of fish from the animal experiment.

| Treatment/  Age Group | Length (cm) | | | Weight (g) | | |
| --- | --- | --- | --- | --- | --- | --- |
|  | Pre | Treatment | Post | Pre | Treatment | Post |
| Control – Ad | 27.3 ± 0.6 | 27 ± 1.09 | 27.8 ± 1.48 | 233 ± 14.55 | 232.6 ± 29.37 | 250.9 ± 42.74 |
| Control – Juv | 15 ± 0.62 | 14.24 ± 1.23 | 12.9 ± 1.19 | 43.8 ± 5.56 | 37.2 ± 11.34 | 29.5 ± 8.2 |
| FF – Ad | 24.8 ± 0.75 | 29.86 ± 2.27 | 27.2 ± 0.68 | 181.2 ± 16.26 | 300.4 ± 59.03 | 253 ± 18.51 |
| FF – Juv | 14 ± 0,63 | 14.4 ± 1.02 | 13.5 ± 0.89 | 31.6 ± 4.59 | 34.6 ± 9.41 | 33.6 ± 5.44 |
| Mixture – Ad | 27.2 ± 1,16 | 29.8 ± 1.6 | 28 ± 2.27 | 253.6 ± 28.71 | 293.6 ± 42.95 | 263.9 ± 60.17 |
| Mixture – Juv | 14 ± 0.64 | 14.4 ± 0.8 | 13.7 ± 0.2 | 32 ± 3.22 | 37.4 ± 5.2 | 36.8 ± 1.77 |
| PAA – Ad | 26 ± 1.09 | 26.92 ± 2.67 | 29.6 ± 1.04 | 216.4 ± 38.15 | 223.4 ± 42.82 | 311.8 ± 19.97 |
| PAA – Juv | 14.4 ± 0.49 | 12.1 ± 1.8 | 14.2 ± 0.24 | 29.2 ± 6.05 | 27 ± 16.03 | 37.6 ± 2.63 |

Mean (+ SD) length and weight measurements for control or treated brown trout before (Pre), during (Treatment) and after (Post) florfenicol (FF) and peracetic acid (PAA) treatment. Ad indicates adult fish, juv indicates juvenile fish.

**Table S2:** Measurements of water parameters throughout the experiment period.

| Treatment/Age Group | diss. oxygen (mg/L) | pH | NH_4_^+^ (mg/L) | Temperature (°C) |
| --- | --- | --- | --- | --- |
| Control – Ad | 7.84 ± 1.15 | 7.84 ± 0.16 | 0.48 ± 0.25 | 11.23 ± 0.42 |
| Control – Juv | 9.40 ± 0.45 | 8.03 ± 0.16 | 0.24 ± 0.15 | 11.08 ± 0.44 |
| FF – Ad | 9.50 ± 3 | 7.96 ± 0.21 | 0.41 ± 0.25 | 11.24 ± 0.37 |
| FF– Juv | 8.86 ± 0.48 | 8.04 ± 0.14 | 0.30 ± 0.20 | 11.07 ± 0.44 |
| Mixture – Ad | 8.95 ± 0.56 | 8.08 ± 0.07 | 0.43 ± 0.23 | 11.13 ± 0.41 |
| Mixture – Juv | 9.33 ± 0.41 | 8.12 ± 0.14 | 0.33 ± 0.17 | 10.89 ± 0.47 |
| PAA – Ad | 8.83 ± 0.42 | 8.05 ± 0.09 | 0.56 ± 0.26 | 11.05 ± 0.47 |
| PAA – Juv | 9.67 ± 0.29 | 8.19 ± 0.18 | 0.48 ± 0.23 | 10.66 ± 0.58 |

Mean (+ SD) concentrations of dissolved oxygen (mg/L), pH, NH_4_^+^ (mg/L) and temperature (°C) in tank water recorded during the experiment. Ad = Adult, Juv = Juvenile, FF = Florfenicol, PAA = Peracetic Acid.

**Table S3:** Composition of commercial feed, supplied from Alltech Coppens, Germany.

|  | Adult | Juvenile |
| --- | --- | --- |
| Product | Supreme-22 | Pre Grower-18 |
| Particle Size | 4.5 mm | 2 mm |
| Protein [%] | 43-45 | 45 |
| Fat [%] | 20-23 | 18 |
| Crude fibre [%] | 1 – 2 | 1.3 |
| Ash [%] | 4 – 8 | 9.3 |
| Total P [%] | 0.82 | 1.5 |

**Table S4:** Two-Way ANOVA output of generalized linear model on ASV richness.

|  | **ASV Richness** | | | |
| --- | --- | --- | --- | --- |
|  | Adult | | Juvenile | |
|  | F-Value | p-Value | F-Value | p-Value |
| Treatment | 0.530 | 0.6635 | 3.565 | **0.0194** |
| Timepoint | 1.610 | 0.1819 | 3.128 | **0.0213** |
| Interaction | 1.998 | **0.0379** | 0.823 | 0.6262 |
| Sex | 0.952 | 0.3328 | nt | nt |

nt = not tested (fish were not sexually mature)

**Table S5:** Topological properties of bacterial co-occurence networks comparing post-treatment networks per age to control networks.

|  | **Control** | | | **Post-Treatment** | | |
| --- | --- | --- | --- | --- | --- | --- |
|  | Adult | Juvenile | Adult | | Juvenile |  |
| Component Size | 676 | 529 | 567 | | 477 |  |
| Clustering coefficient | 0.0908 | 0.0876 | 0.1001 | | 0.0996 |  |
| Modularity | 0.2951 | 0.3389 | 0.2937 | | 0.3104 |  |
| Pos. Edges [%] | 72.05 | 74.44 | 61.47 | | 63.53 |  |
| Edge density | 0.0269 | 0.0225 | 0.0262 | | 0.0260 |  |
| Nat. connectivity | 0.0032 | 0.0032 | 0.0032 | | 0.0035 |  |
| Av. path length | 2.6395 | 2.9605 | 2.7428 | | 2.8137 |  |
| No. of Edges | 6135 | 3142 | 4199 | | 2956 |  |
| No. of Clusters | 5 | 8 | 7 | | 9 |  |
| Av. Betweenness | 553.32 | 517.56 | 493.22 | | 431.67 |  |
| Av. Degree | 38.30 | 25.76 | 31.62 | | 26.78 |  |

All treatment samples sampled at timepoint 28 (18 days post-treatment) are combined into one post-treatment network per age group.
