# Supplementary material for: Age matters: exploring differential effects of antimicrobial treatment on gut microbiota of adult and juvenile brown trout (Salmo trutta)
Source: Anim Microbiome. 2025 Mar 16;7:28. doi: 10.1186/s42523-025-00391-2 (PMC11910850; doi:10.1186/s42523-025-00391-2)
Supplement: Supplementary file 2 — Additional file 2. [file 42523_2025_391_MOESM2_ESM.docx]

**Additional File 2 for „Age Matters: Exploring differential effects of antimicrobial treatment on gut microbiota of adult and juvenile brown trout (*Salmo trutta fario*), containing supplementary figures**

**Figure S1:** Rarefaction curve showing observed ASV richness of all samples analyzed in this study. light green = juvenile, dark green = adult.

**Figure S2:** Observed ASV Richness in gut microbial communities of juvenile and adult brown trout, comparing unexposed control groups to either florfenicol or peracetic acid-treated groups before, during and after the treatment.

**Figure S3:** Compositional heatmap representing core ASVs in the gut microbiome of (a) adult and (b) juvenile brown trout. Prevalence = 70 %, detection threshold = 0.001.

**Figure S4:** Heatmap showing the relative abundance of highly influential hub ASVs (IVI score > 50) on genus level in control and post-treatment co-occurrence networks of both age groups. Ad = adult, juv = juvenile.
